# Fluoridation cessation: More science from Alberta

**DOI:** 10.1111/cdoe.12346

**Published:** 2017-10-10

**Authors:** Lindsay McLaren, Steve Patterson, Salima Thawer, Peter Faris, Deborah McNeil, Melissa Potestio

**Affiliations:** ^1^ University of Calgary Calgary AB Canada; ^2^ University of Alberta Calgary AB Canada; ^3^ Alberta Health Services Calgary AB Canada

**Keywords:** community water fluoridation, public health, research

Thank you for the opportunity to respond.^1^ We are pleased to see thoughtful debate in the peer‐reviewed literature and agree that careful consideration of study limitations can stimulate improvement.

## 1 AVAILABLE DATA

As with many large‐scale research projects, we had more information than was possible to include in a single paper. We thus prepared multiple papers addressing different aspects of the study: overall trends in dental caries—tooth‐level data^2^ and tooth surface‐level data;^3^ and trends by socioeconomic indicators^4^ (Neurath et al [“the authors”] erroneously state that we “control” for these in the latter paper).

The authors focus on a single paper.^3^ We did not include the 2009/2010 data point in that paper because of its focus on tooth surface‐level data, which the 2009/2010 data point did not include.

Considering our whole work,^2^, ^3^, ^4^ one will find the data point in question, including our observation in Calgary between 2004/2005‐2009/2010 (precessation) and 2009/2010‐2013/2014 (largely postcessation) of a small increase in slope for *deft* prevalence (%>0; the worsening speeds up) in the latter relative to the former. We reasoned^2^ that fluoridation cessation might first affect prevalence rather than means, which are influenced by children with more severe caries.

Importantly, we highlighted^2^ reasons why comparison across the three Calgary data points must be undertaken with caution, including absence of a 2009/2010 Edmonton data point, which, coupled with the wide confidence interval in Calgary, makes it problematic to draw conclusions from that data point.

## 2 CONSIDERATION OF CONFOUNDING

The authors erroneously state that we included a comparison community instead of measuring potential confounders. In fact, we collected socio‐demographic and behavioural data as part of our 2013/2014 survey. We computed caries estimates adjusting for differences between the Calgary and Edmonton samples and showed that estimates did not materially change.^2^ This suggests that postcessation caries estimates were not an artefact of sample differences. The precessation surveys were part of surveillance activities and did not include a questionnaire. We therefore could not examine differences in baseline (or changes) in those variables. That is an important limitation, which we acknowledged.^2^, ^3^

We considered several potential confounders, including sealants and public health programs.^2^ None provided strong alternative explanations.

The authors correctly note that our outcome assessment was not blind and could have some bias. In our 2013/2014 survey, we collected fingernail clippings from a small random subsample (n = 35) in each city. Total fluoride intake based on those biomarkers, blind to city and fluoridation status confirmed substantially lower fluoride in Calgary (cessation) than in Edmonton (still fluoridated).^2^

## 3 COMPARISON CITY

One could study the effects of fluoridation cessation by observing one community over time, as some have performed.^5^ Our design is strengthened by including a comparison community. In Alberta, there is no better comparison community for Calgary than Edmonton. The authors refer to a “control” community, which is erroneous because it implies that fluoridation cessation was a research intervention.

## 4 PARTICIPATION RATES, POSSIBLE SELECTION BIASES

The authors raise concern about low participation rates, which they erroneously conflate with selection bias (one can have low, but representative, participation).^6^ As with any voluntary survey, some bias may exist, but we found no obvious patterns by school system, income quartile or geographic area. To help produce unbiased estimates of population values, we took the well‐established approach of developing sampling weights,^7^ which incorporated weights for the primary sampling unit (school) and poststratification weights for socioeconomic status (after‐tax median household income of the dissemination area in which the child's school was located).

## 5 SUBGROUP ANALYSES AND BOTTLED WATER CONSUMPTION

The authors described our assessment of smooth tooth surfaces and of children with some tooth decay, as “subgroup analysis.” We had good reasons for these assessments. We examined trends focusing only on smooth surfaces because these are most likely to be affected by fluoridation for the age group and time frame studied. We examined trends among children with some (>0) decay because decay experience in the population is skewed.

The commentary did not mention our analysis of permanent teeth. The observed tooth‐level decrease (improvement) in permanent decay in Calgary^2^ was muted when focusing on smooth‐surface caries only; for mean *DMFS* among those with *DMFS *> 0, the direction of change became positive, although not statistically significant. We reasoned^3^ that this could be an early hint of an adverse effect of fluoridation cessation on permanent tooth caries, but confirmation needs additional monitoring.

Increased bottled water consumption was one of several possible explanations offered for the increase in primary tooth caries. Perhaps more relevant for this was our analysis of lifelong residents who report usually drinking tap water. If there is an effect of fluoridation cessation on dental caries, it should be stronger in this subsample. Although estimates were based on small numbers, and again we are limited by the absence of this information at precessation, they were consistent with an apparent increase in permanent tooth smooth‐surface caries in Calgary: the 2013/2014 Calgary estimate of mean *DMFS* among those with *DMFS *> 0 was higher in the subsample (that is, even more discrepant from the 2004/2005 estimate) than in the full sample.^3^

## 6 STUDY DESIGNS TO ASSESS FLUORIDATION EFFECTIVENESS

The authors argue that randomized controlled trials (RCTs) are needed, a comment which neglects that this population‐level measure is not under researcher control. While one might theoretically envision a cluster‐randomized design,^8^ the unit of randomization would have to be community, not (as they state) households, in respect of the level of intervention.

They also assert (unreferenced) that “next in order of quality, after RCTs, is the longitudinal study with individual‐level information on the same subjects over time.” Instead, one might argue that a next‐best design would incorporate an individual‐level longitudinal component (to study individual‐level trajectories) and a cross‐sectional time‐series component (to compare children of the same age at different times). These are different but equally important questions here. One model is the British birth cohort studies, with staggered cohorts of individuals.^9^

While we agree with the value of stronger designs, one must be thoughtful about evaluation of public health measures, which by definition are complex and context‐dependent.^8^ We used the best available data and design for our circumstances.

## 7 CONCLUSIONS

Studies of fluoridation cessation and dental caries are few in number, highly diverse in time and place, and variable in quality.^5^ Our research improves on limitations of previous studies, and we anticipate that future studies will improve on ours.
